# Altered gut microbiota correlates with cognitive impairment in Chinese children with Down’s syndrome

**DOI:** 10.1007/s00787-021-01799-2

**Published:** 2021-05-17

**Authors:** Shimeng Ren, Xinjuan Wang, Jiong Qin, Qing Mu, Shuai Ye, Yang Zhang, Weidong Yu, Jingzhu Guo

**Affiliations:** 1 Department of Pediatrics, Peking University People’s Hospital, Beijing, 100044 China; 2 Department of Central Laboratory & Institute of Clinical Molecular Biology, Peking University People’s Hospital, Beijing, 100044 China

**Keywords:** Gut microbiota, Down’s syndrome, Cognitive impairment, 16S rRNA sequencing

## Abstract

Down's syndrome (DS), a common chromosomal disease caused by chromosome 21 trisomy, is the main cause of cognitive impairment in children worldwide. Emerging evidence suggests that the microbiota–gut–brain axis plays a potential role in cognitive impairment. However, data regarding gut microbiota alterations in DS patients remain scarce, especially data from children with DS. This case–control study was conducted to explore the gut microbiota composition in Chinese DS children. Additionally, the potential association between gut microbiota and cognitive function in DS was evaluated. Microbiota communities in the feces of 15 DS subjects and 15 matched controls were investigated using high-throughput Illumina Miseq sequencing targeting the V3–V4 region of 16S rRNA gene. The relationships between gut microbiota composition and DS cognitive function scores were analyzed. The structure and richness of the gut microbiota differed between DS patients and healthy controls. The abundance of *Acidaminococcaceae* was decreased in DS patients. Moreover, the Kyoto Encyclopedia of Genes and Genomes analysis showed increased modules related to peptidases and pyrimidine metabolism. Overall, we confirmed that gut microbiota alterations occurred in Chinese patients with DS. Additionally, the fecal microbiota was closely related to DS cognitive impairment. Larger cohorts are needed to confirm these findings and to clarify the mechanisms involved. Elucidating these novel findings in the field of microbiota-gut-brain axis will provide a promising strategy for future studies of DS cognitive impairment.

**Supplementary Information:**

The online version contains supplementary material available at 10.1007/s00787-021-01799-2.

## Introduction

Down's syndrome (DS) is the most common chromosomal disease in China. It occurs in approximately 26,600 births annually, resulting in an estimated prevalence of 13 per 10,000 live births [1]. Around 5–8 million people are living with DS-associated cognitive impairment worldwide, and about 70% of them have progressive dementia associated with Alzheimer's disease (AD) after the age of 40 [2]. As there is no effective medical therapy to improve cognitive function or delay the occurrence of AD-related dementia, DS patients cannot function normally, which results in a heavy burden to their families and society [3].

DS is caused by chromosome 21 trisomy. Chromosome 21 contains hundreds of genes, including the gene for amyloid precursor protein [4]. Accumulation of amyloid-β (Aβ), a cleavage product of amyloid precursor protein gene, in the brain can trigger microglial activation and neuron damage in AD patients [5, 6]. Since DS patients have three copies of the amyloid precursor protein gene, DS is associated with a significant increase in the risk of early AD-related dementia [7]. Prior to Aβ plaque formation, higher levels of plasma Aβ40 and Aβ42 were founded in the brains of children with DS [8]. Meanwhile, Aβ-induced inflammatory cell activation can affect brain growth in DS, suggesting that some common pathogenic mechanisms may exist between DS-associated cognitive impairment and AD [9, 10].

Recent studies have shown that alterations in the gut microbiome [11, 12] may play a role in the pathogenesis of cognitive impairment in AD [13, 14]. Additionally, studies have found that gut microbiota alterations in AD affect cognitive function by altering the status of the microglia [15]. Although this evidence indicates a pathogenic link between gut microbiota and cognitive function, few studies have explored the role of the gut microbiota in DS-associated cognitive impairment. Additionally, these studies have not been carried out in pediatric DS patients or in the Chinese population. A recent study from Italy using 16S rRNA gene sequencing showed that adult patients with DS exhibit gut microbiota alterations [16]. However, there are major differences in the bacterial profiles of the gut between adults and children in different populations. Childhood is a key stage of cognitive development [17]. In addition, increasing evidence has shown that age, geographic origin, and eating habits have a great influence on the composition of the gut microbiota [18, 19].

Therefore, in our study, we utilized a healthy control group consisting of children who attended the same school as the DS patients for at least 2 years. Their ages, geographic origins, and diets were well matched to those of the DS patients. We aimed to compare the microbiota communities in the feces of DS subjects to those of the matched controls, and the results of this study may provide a new target for future studies of cognitive impairment in DS.

## Materials and methods

### Study subjects and ethical approval

The study protocol was approved by the Ethical Review Committee of Peking University People’s Hospital, Beijing, China (approval no. 2019PHB110-01). All participants and their parents received verbal and documented information on the study, and written consent was obtained prior to recruitment.

**Fig. 1:**
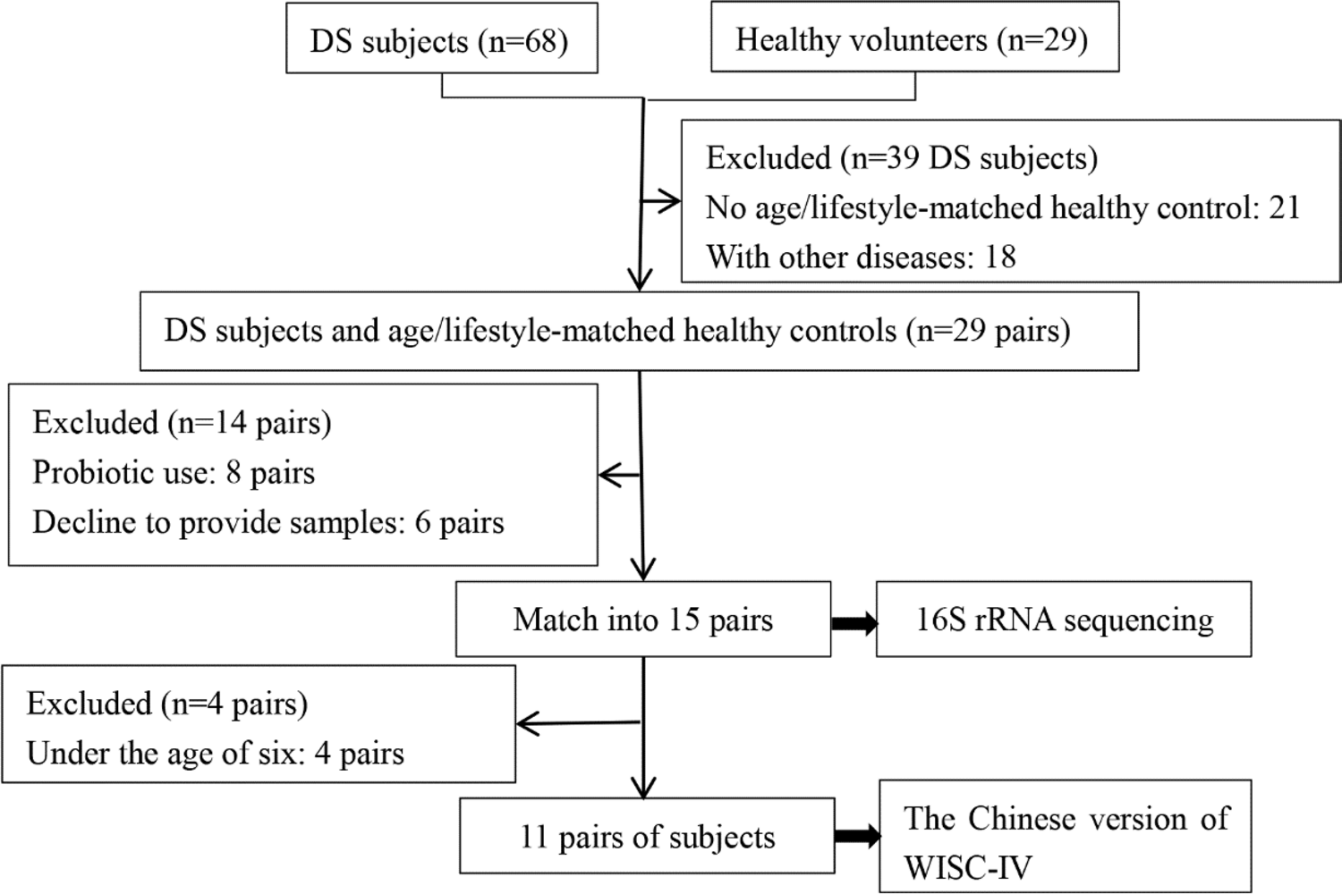
Flow chart illustrating the recruitment of DS and healthy subjects

We randomly recruited 68 DS children and 29 healthy volunteers from the boarding integrated school in Beijing and its surrounding areas. All the study subjects had the same diet offered by school. 18 DS children with other diseases and 21 DS subjects who have no age-matched healthy controls were excluded. Among the remaining 29 pairs of subjects, each pair consists of a DS subject and an age-matched healthy control. Additionally, 14 pairs were excluded due to the use of probiotics and refusal to provide samples (detail information provided in supplementary Fig. S1). A total of 15 pairs of subjects were included in the final analysis of 16S rRNA sequencing (recruitment flow chart provided in Fig. 1). 11 pairs of participants over the age of 6 were tested with the Chinese version of the Wechsler Intelligence Scale for Children (WISC-IV) [20]. Given that the cognition test of WISC-IV is only applicable to children over the age of 6, 4 pairs of participants under the age of 6 were excluded.

A chromosome test was used to establish a diagnosis of DS, and the healthy controls exhibited no disease symptoms. Exclusion criteria for participants included a history of nutritional supplement use and special diets, presence of significant physical abnormalities, and neurological disorders of known etiology. Individuals who took antibiotics or probiotic supplements within the three months prior to sample collection were also excluded.

### Clinical data collection

Each subject’s weight and height were measured, and their body mass index was calculated. Participants were interviewed by a nurse or resident regarding their bowel habits. Gastrointestinal symptoms were assessed following a constipation scoring system adapted for children. The constipation scoring system, also known as the Cleveland Clinic score, was proposed by Agachan et al. to assess the severity of intestinal dysfunction [21]. It includes frequency of bowel movements, painful evacuation efforts, feeling incomplete, abdominal pain, minutes in lavatory per attempt, type of assistance, unsuccessful evacuation attempts per 24 h, and duration of constipation. The scores ranged from 0 to 30, with 0 indicating normal bowel function and 30 indicating severe constipation. A score of 15 or more is used to define "constipation" in this scoring system.

For the purposes of the present study, we examined the scores obtained in the 10 core subtests of the WISC-IV: block design, similarities, digit span, picture concepts, coding, vocabulary, letter-number sequencing, matrix reasoning, comprehension, and symbol search. We calculated the Full scale IQ (FSIQ) and the four factor indices: the perceptual reasoning index (PRI), which includes block design, picture concepts, and matrix reasoning; the verbal comprehension index (VCI), including similarities, vocabulary and comprehension; the working memory index (WMI), including digit span and letter-number sequencing; and the processing speed index (PSI), including coding and symbol search. Cognitive tests were standardly administered by two trained pediatricians at hospital meeting room that were free of distractions. The intra-class correlation coefficient was about 0.857.

### Fecal sample collection and DNA extraction

Fecal samples were collected using fecal bacteria DNA storage tubes (Longsee Biological Company, Guangzhou, China). The samples were transferred on ice and stored at − 80 ℃ prior to processing. The average transfer time between sample collection and stored at − 80 ℃ was 19 min, and the maximum time was 23 min. Total fecal DNA was extracted using a QIAamp DNA Stool Mini Kit (Qiagen, Hilden, Germany). All DNA extractions were performed in a Class II biological safety cabinet. The concentration of genomic DNA in each fecal sample was quantified using a Nano-Drop 2000 spectrophotometer (Thermo Scientific, MA, USA). DNA integrity and sizes were assessed by 1% agarose gel electrophoresis. The DNA was re-suspended in H_2_O and stored at − 80 ℃ prior to use.

### 16S rRNA gene amplicon and sequencing

The isolated bacterial genomic DNA was used as a template for PCR amplification of V3-V4 region of the bacterial 16S rRNA gene in a multiplex approach with the forward primers and the reverse primer. The amplicons were extracted and further purified using the AxyPrep DNA Gel Extraction Kit (Axygen Biosciences, Union City, CA, USA) and quantified using the QuantiFluor™-ST system (Promega, USA) per the manufacturer’s instructions. The sequencing data were pooled equimolarly and paired-end sequenced (2 × 300) on an Illumina MiSeq platform (Illumina, San Diego, USA) according to standard protocols from Majorbio Bio-Pharm Technology Co. Ltd. (Shanghai, China). We obtained 1,851,666 sequences and 763,428,366 bases from 30 samples with an average of 61,722 sequences per sample. The average length of sequences was about 412 bases (amplify region, 338F_806R) and length distribution of sequences is shown in Fig. S2. Detailed descriptions of the amplicons and the sequencing analysis protocol were provided in the eMethods in the Supplementary data.

### Statistical analysis

SPSS (ver. 20.0, SPSS Inc., Chicago, IL, USA) and R software (ver. 3.1.0, the R Project for Statistical Computing) were used for statistical analysis. Comparisons between groups were assessed using the Student’s *t* test or the Wilcoxon rank-sum test for quantitative variables and Pearson’s Chi-square test for categorical variables, respectively. Rarefaction was applied to the operational taxonomic units (OTUs) to reduce sampling heterogeneity for further alpha and beta-diversity calculations. Partial least-squares-latent structure discriminant analysis (PLS-DA) was performed to compare the fecal microbiota structures in different groups based on OTUs from the sequencing data of each sample. The principal coordinate analysis (PCoA) of weighted Unifrac distance matrices was performed to measure the β-diversity. Differential abundance analysis was performed using the Wilcoxon rank-sum test at the class, order, family, genus, and species levels. And the false discovery rate was calculated using the Benjamini–Hochberg method. The linear discriminant analysis (LDA) effect size (LEfSe) analysis, an algorithm for high-dimensional biomarker discovery, was used to identify differentially abundant taxa between two groups. The LEfSe method was used to estimate the effect of each differentially abundant taxon and discriminate the most biologically relevant taxon.

Random forest analysis was used to predict the disease status based on the microbiota profile (genus and OTU-level relative abundance data) using the default parameters of the R implementation of the algorithm (R random forest package). Bootstrapping (*n* = 500) was used to assess the classification accuracy. To evaluate the discriminatory ability of the random forest model, receiving operational curves (ROC) were constructed and the area under curve (AUC) was calculated. Additionally, Phylogenetic Investigation of Communities by Reconstruction of Unobserved States (PICRUSt) was used to predict the abundance of functional categories, such as KEGG pathways, based on the 16S rRNA sequence data. The Principal component analysis (PCA) colored by FSIQ score was also performed (intergroup difference test method: ANOSIM, permutations = 999). The coloring order of the label scale was the order of FSIQ levels. Spearman correlation and MaAslin analysis were used to assess the relationship between bacterial taxa and WISC-IV scores. *p* < 0.05 was considered statistically significant.

## Results

### Characteristics of the DS and healthy groups

The demographic characteristics of the DS group and the healthy group are summarized in Table 1. There were no differences in age, gender, or weight between the two groups. The DS group had a lower average height (*p* = 0.037) and higher body mass index (*p* = 0.009) than the healthy group (Table 1). A higher proportion of the DS group patients reported constipation compared to the healthy group (33.3% vs 0%, *p* = 0.042, Table 1). Significant differences were found in frequency of bowel movements (*p* = 0.043), feeling incomplete evacuation (*p* = 0.043), abdominal pain (*p* = 0.009), minutes in lavatory per attempt (*p* = 0.03), type of assistance (*p* = 0.028), and duration of constipation (*p* < 0.001). The DS group had lower FSIQ, VCI, PRI, WMI, and PSI scores compared to the healthy group (all *p* < 0.001, Table 2).

**Table 1:** Characteristics of the study subjects

Characteristics	DS group (*n* = 15)	Healthy group (*n* = 15)	*p* value	*t* value	95% CI
Age(years)^a^	6.96 (1.98)	6.82 (1.97)	0.85	0.194	− 1.34 to 1.622
Gender (*n*, %)			0.713	–	–
Male	9 (60%)	8 (53.33%)		–	–
Female	6 (40%)	7 (46.67%)		–	–
Height (m)^a^	1.1 (0.15)	1.22 (0.14)	0.037	− 2.188	− 0.23 to − 0.008
Weight (kg)^a^	19.69 (5.62)	23.17 (5.57)	0.1	− 1.702	− 7.66 to 0.707
Body mass index (kg/m^2^)^a^	15.9 (0.72)	15.33 (0.31)	0.009	2.797	0.143 to 0.992
Constipation (*n*, %)^a^	5 (33.33%)	0 (0%)	0.042	–	–
Frequency of bowel movements^a^	0.8 (0.78)	0.27 (0.59)	0.043	2.117	0.017 to 1.049
Painful evacuation effort^a^	1.33 (0.72)	0.93 (0.8)	0.162	1.437	− 0.17 to 0.97
Feeling incomplete evacuation^b^	1.8 (0.86)	1.27 (0.46)	0.043	2.117	0.009 to 1.057
Abdominal pain^a^	2.2 (1.2)	1.2 (0.68)	0.009	2.799	0.268 to 1.732
Minutes in lavatory per attempt^b^	1.73 (0.88)	1.07 (0.7)	0.03	2.286	0.069 to 1.264
Type of assistance^b^	1.33 (0.49)	0.93 (0.46)	0.028	2.316	0.046 to 0.754
Unsuccessful attempts for evacuation per 24 h^a^	1 (0.85)	0.87 (0.74)	0.65	0.459	− 0.46 to 0.729
Duration of constipationa^b^	1.13 (0.35)	0.07 (0.26)	< 0.001	9.466	0.836 to 1.297

Data are shown as mean (SD)^a^ or median (IQR)^b^

*DS* Down’s syndrome, *CI* confidence interval

^a^*SD* standard deviation

^b^*IQR* interquartile range

**Table 2:** Cognitive function scores assessed according to Chinese version the WISC-IV

	DS group (*n* = 11)	Healthy group (*n* = 11)	*p* value	*t* value	95% CI
Full scale IQ, FSIQ^a^	55.18 (6.21)	100.36 (10.78)	< 0.001	− 12.051	− 53.002 to − 37.361
Verbal comprehension index, VCI^a^	60.64 (8.56)	98.73 (10.73)	< 0.001	− 9.202	− 46.725 to − 29.456
Perceptual reasoning index, PRI^b^	51.18 (4.05)	98.82 (11.09)	< 0.001	− 13.385	− 55.06 to − 40.213
Working memory index, WMI^a^	49.54 (3.64)	102.9 (12.7)	< 0.001	− 13.396	− 61.673 to − 45.054
Processing speed index, PSI^a^	56.09 (6.77)	100.3 (9.86)	< 0.001	− 12.273	− 51.798 to − 36.748

Data are shown as mean (SD)^a^ or median (IQR)^b^

*DS* Down’s syndrome, *CI* confidence interval

^a^*SD* standard deviation

^b^*IQR* interquartile range

### Alpha and beta-diversity between the DS and healthy groups

**Fig. 2:**
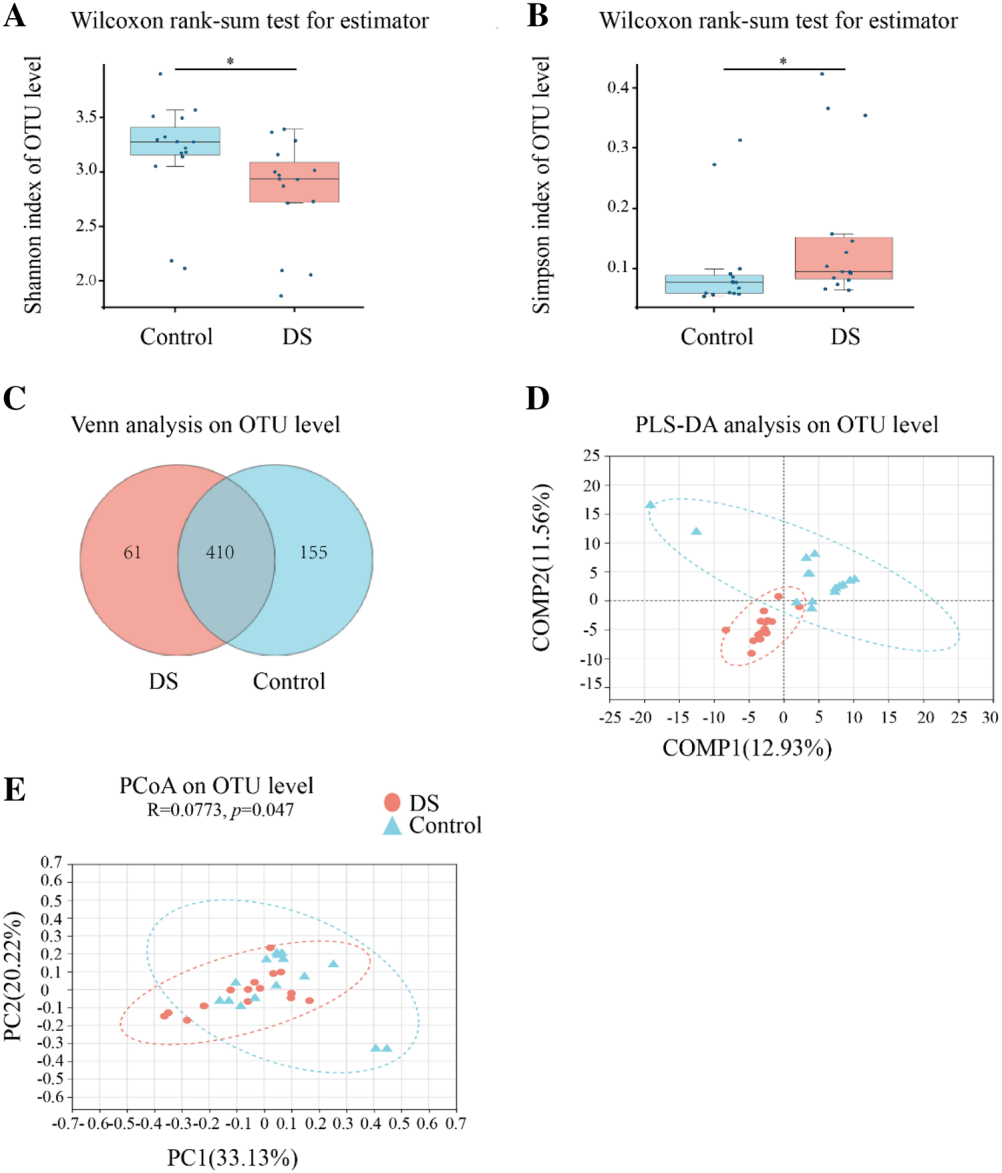
Comparison of the fecal microbiota composition between the DS and Healthy control (HC) groups. **a**, **b** Alpha diversity based on the Shannon index and Simpson index at the OTU level. Wilcoxon rank-sum test, Control vs. DS, *p = 0.01; **c** Venn diagram illustrating the overlap of the OTUs identified in the fecal microbiota between the two groups. **d** PLS-DA score plots based on the relative abundances of microbiota between the two groups. **e** The principal coordinate analysis (PCoA) on the OTU level. The comparison of β-diversity between DS and healthy group was performed by PCoA with weighted Unifrac distance matrices, ANOSIM, permutations: 999, *R* = 0.0773, *p* = 0.047. *p < 0.05 vs. HC group

To assess the alteration of the fecal microbiota composition in the DS group, we performed parallel Miseq sequencing. As shown in Fig. 2a, the Shannon diversity index was significantly reduced in the DS group (control: 3.18 vs. DS: 2.82, *p* = 0.01), whereas the Simpson’s diversity index (control: 0.1 vs. DS: 0.16, *p* = 0.01) was increased (Fig. 2b), suggesting decreased commensal diversity in patients with DS. As shown in the Venn diagram, 410 out of 626 total OTUs were shared among all the samples (Fig. 2c). The distinctive fecal microbial communities associated with DS are presented in a supervised PLS-DA plot (Fig. 2d). Significant differences were also found in the beta-diversity, indicating that the fecal microbial structure in the DS group differed significantly from that of healthy group (Fig. 2e).

### Alteration in taxa abundance between the DS and healthy groups

We assessed the mean relative abundances of various taxa in the DS and healthy groups to identify specific alterations in the microbiota. 9 bacteria taxa were identified in supplementary Table S1 (*p* < 0.05). After adjustment by the Benjamini–Hochberg method, only proportions of family *Acidaminococcaceae* were different between two groups (*q* = 0.04001, 95% confidence interval: − 1.819 to 1.445, effect size = 0.1965, Fig. 3a, b).

**Fig. 3:**
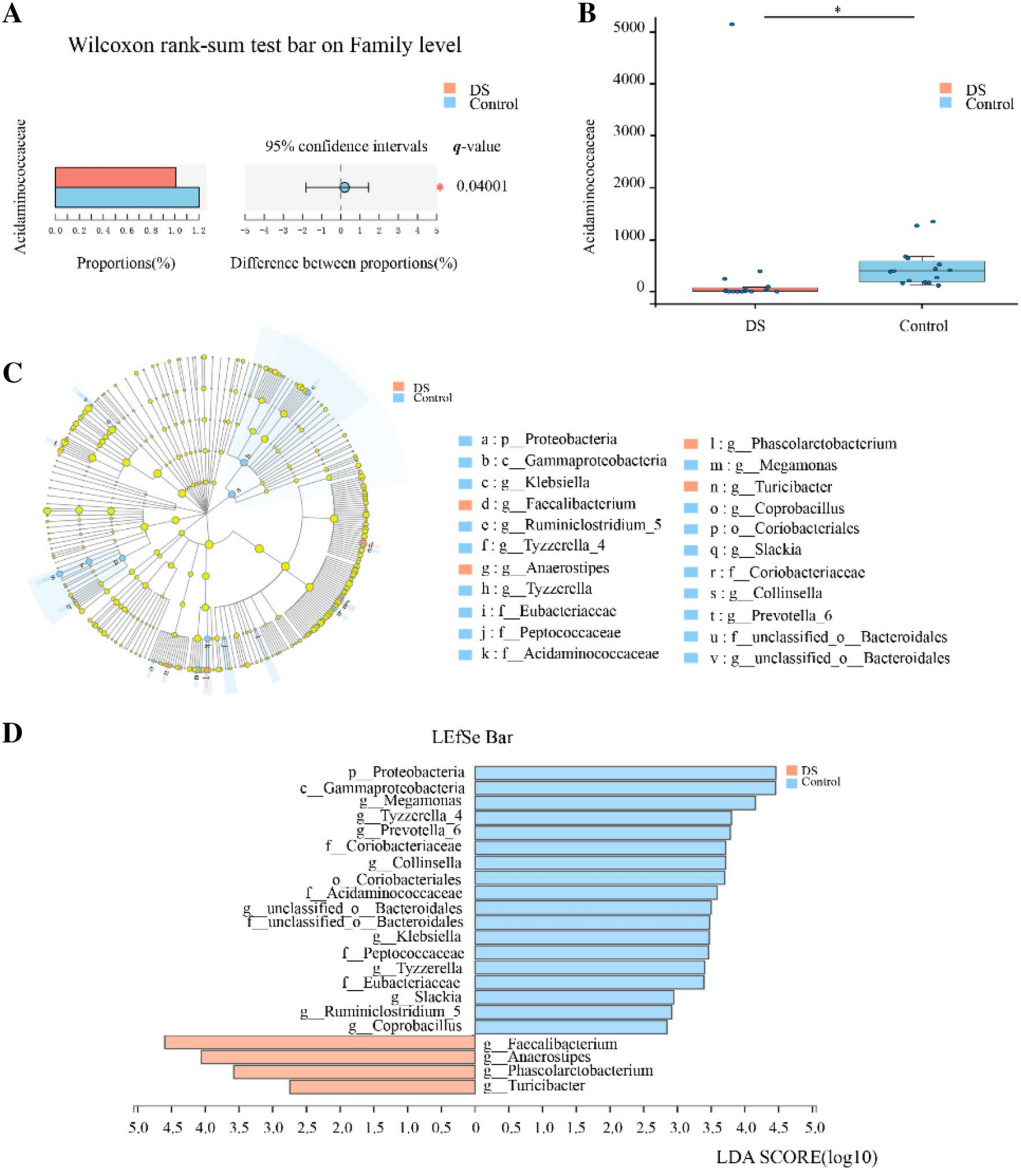
Abundances of taxa in DS and HC participants. **a**, **b** Wilcoxon rank-sum test of the mean relative abundances at “Family” level in DS and HC participants. The significantly different taxa after adjusting by the Benjamini–Hochberg method were shown in *q* value. Red and blue boxplot indicate the mean relative abundances of taxa in the DS and Control subjects, respectively. *q < 0.05 vs. HC group. **c**, **d** LEfSe analysis of the bacterial taxa associated with the DS and HC groups. The Cladogram generated by the LEfSe analysis (from phylum to genus level) and LDA scores (genus level) identify differentially abundant bacterial taxa associated with DS and HC subjects. **c** Red and blue dots indicate the bacterial taxa enriched in DS and HC subjects, respectively. **d** Enriched bacterial taxa in DS patients have positive LDA scores (red), and HC enriched bacterial taxa have negative scores (blue). Only taxa having an LDA > 2.0 were shown significant in the figure

To assess the impact of digestive problems on the differences found in microbiota, we divided the DS group into DS_C group (DS with constipation, *n* = 5) and DS_NC group (DS without constipation, *n* = 10). Kruskal–Wallis H test was performed between healthy control group, DS_C group and DS_NC group. No significant difference in microbiota was found after adjusted by the Benjamini–Hochberg method (Fig. S3). To observe the effect of constipation on DS gut microbiota, we have made a further analysis between DS_C group and DS_NC group. There was no significantly different abundance on genus level between two groups (Fig. S4). It needs more large-scale studies to confirm these results in the future.

We performed a supervised comparison of the microbiota between the DS and healthy groups using the LEfSe analysis, which is often used to identify the presence and effect size of region-specific OTUs among different groups. We used a logarithmic LDA score cut-off of 2.0 to identify important taxonomic differences between the DS and healthy groups. In particular, we examined differences in the taxa at the genus level. The results indicated a remarkable difference in the fecal microbiota between the DS and healthy groups. The relative abundances of the genera *Megamonas*, *Tyzzerella_4*, *Prevotella_6*, *Collinsella*, *Klebsiella*, *Slackia*, *Ruminiclostridium_5*, and *Coprobacillus* were higher in the healthy group than in the DS group, whereas the relative abundances of genera *Faecalibacterium*, *Anaerostipes*, *Phascolarctobacterium*, and *Turicibacter* were higher in the DS patients than in the healthy controls (LDA score (log10) > 2, Fig. 3c, d).

### Random forest predictive models

**Fig. 4:**
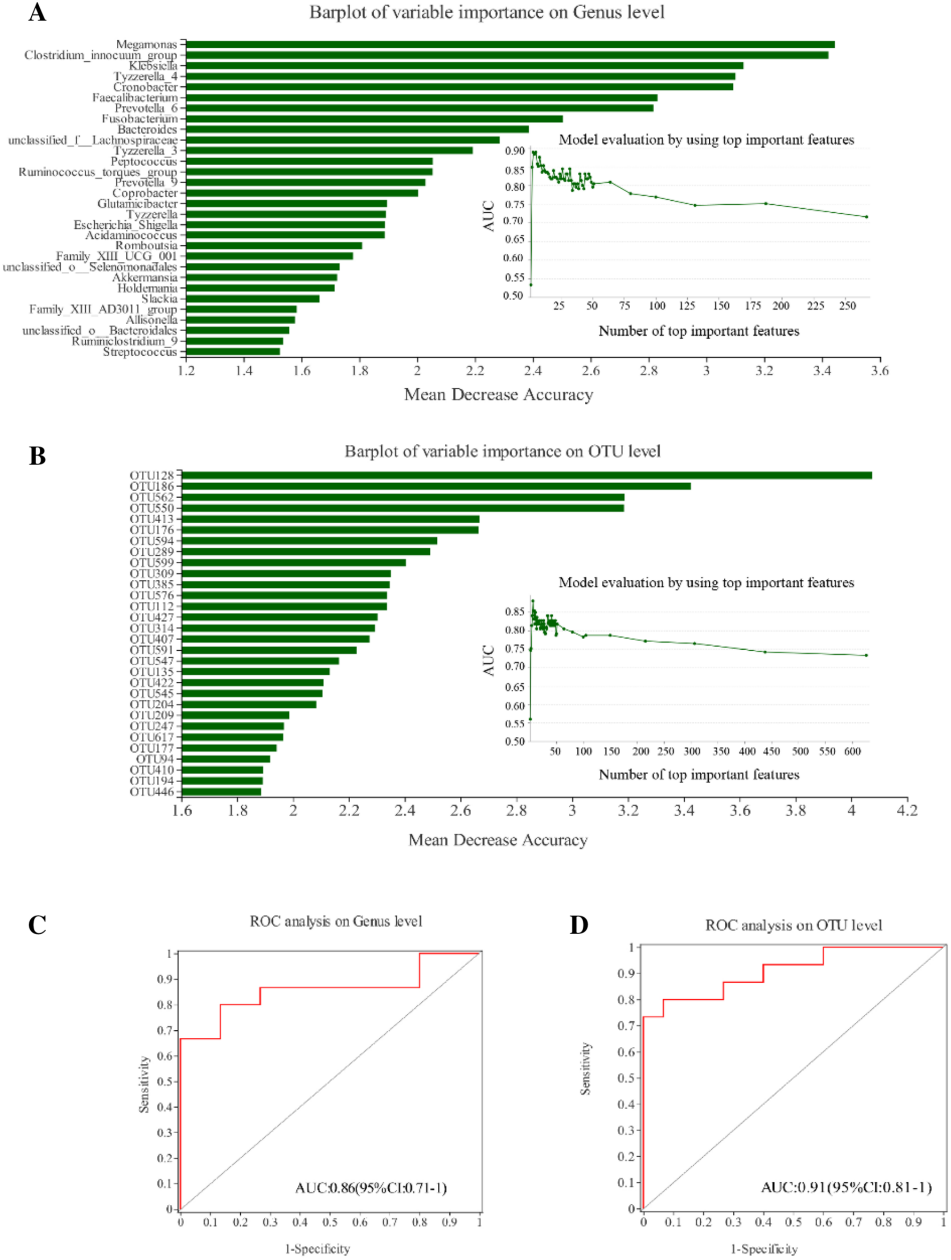
Predictive random forest model based on genus and OTUs. The relative importance of each genus (**a**) and OTU (**b**) in the predictive model was assessed using the mean decreasing accuracy for fecal microbiota. ROC curve generated by the random forest model using 3 genera (**c**) and 6 OTUs (**d**) in the fecal microbiota. AUC marked in the figure is the area under the ROC curve; AUC is usually between 1.0 and 0.5. The closer AUC is to 1, the better the diagnostic effect. When AUC is above 0.9, there is high accuracy of the diagnostic model; When AUC is between 0.7 and 0.9, there is certain accuracy; When AUC is between 0.5 and 0.7, the accuracy of the diagnostic model is low. When AUC = 0.5, it indicates that the diagnostic method is completely ineffective

To evaluate DS disease status based on an ensemble of decision trees, we used random forest analysis based on AUC verification method to build a predictive model at the OTU and genus levels (Fig. 4a, b). According to the point with the highest AUC value, 3 genera and 6 OTUs were selected to construct the random forest model. ROC curves were calculated using the predominant fecal microbial taxa, including 3 genera and 6 OTUs. At the genus level, the area under the ROC curve was 0.86 (95% confidence interval 0.71–1, Fig. 4c), indicating that the diagnostic model had certain accuracy. At the OTU level, the area under the ROC curve was 0.91 (95% confidence interval 0.81–1, Fig. 4d), indicating that the diagnostic model had high accuracy.

### Predictive function analysis

**Fig. 5:**
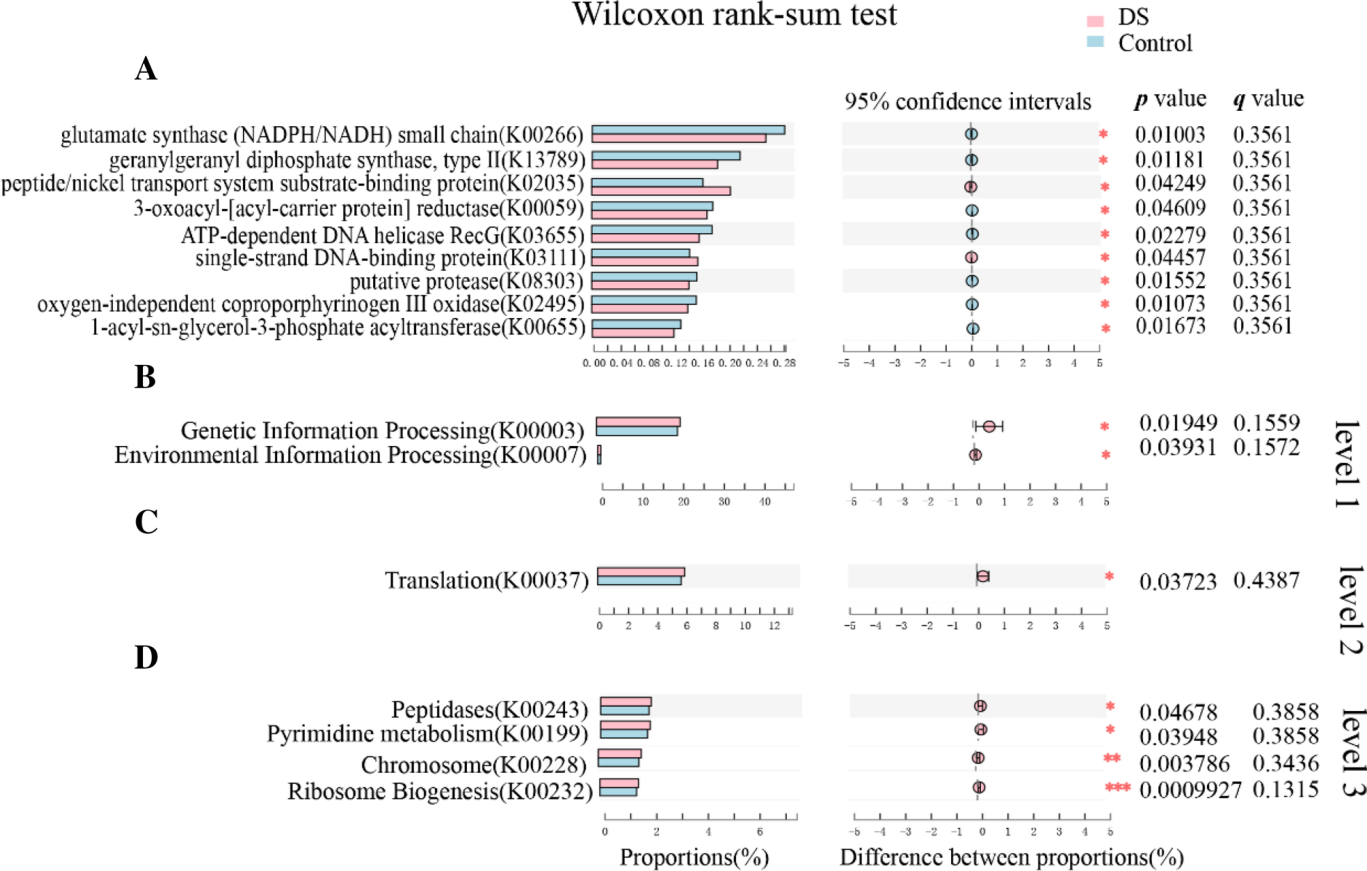
Functional predictions for the fecal microbiomes of the DS and HC groups. **a** KOs with significantly different abundances in the fecal microbiome. Significantly enriched KEGG pathways at level 1 (**b**), level 2 (**c**), and level 3 (**d**) in the fecal microbiome of the DS and healthy groups. Wilcoxon rank-sum test, the false discovery rate (FDR) was calculated using the Benjamini–Hochberg method. Red and blue bars indicate the mean relative abundances of KEGG pathways in the DS and HC subjects, respectively. *p < 0.05 vs. HC group; **p < 0.01 vs. HC group; ***p < 0.001 vs. HC group

PICRUSt based on the closed-reference OTUs was used to predict the abundances of functional categories of the KEGG orthology (KO). A total of 9 KOs in the fecal microbiome between the DS and healthy groups were identified (Fig. 5a). In the level 1 KEGG pathway, the environmental information processing and genetic information processing functions were higher in the fecal microbiome of the DS group (Fig. 5b). In level 2, the pathway involved in translation of genetic information processing was higher in the DS group (Fig. 5c). In level 3, pathways involved with ribosome biogenesis in translation, replication and repair of chromosome in genetic information processing, pyrimidine metabolism of nucleotide metabolism, and peptidases of enzyme families in metabolism were higher in the fecal microbiome of the DS group (Fig. 5d). Larger-scale studies are needed to confirm our findings, considering that the FDR-adjusted results were not statistically significant.

### Association between fecal microbiota and cognitive function

**Fig. 6:**
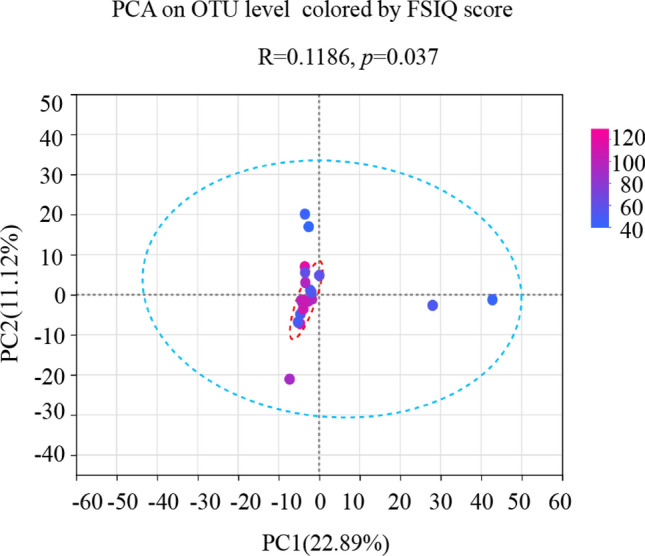
The principal component analysis (PCA) on the OTU level colored by FSIQ score. Intergroup difference test method: ANOSIM, permutations = 999. *X* axis and *Y* axis represent two principal components. Percentage represents the degree of explanation of principal component to the difference in sample composition. The scales on the *X* and *Y* axes are relative distances. A comparison based on the cut-off score has been performed. The coloring order of the label scale is the order of the FSIQ levels. As the FSIQ score decreases, the sample points gradually change from pink to purple and finally to blue

**Fig. 7:**
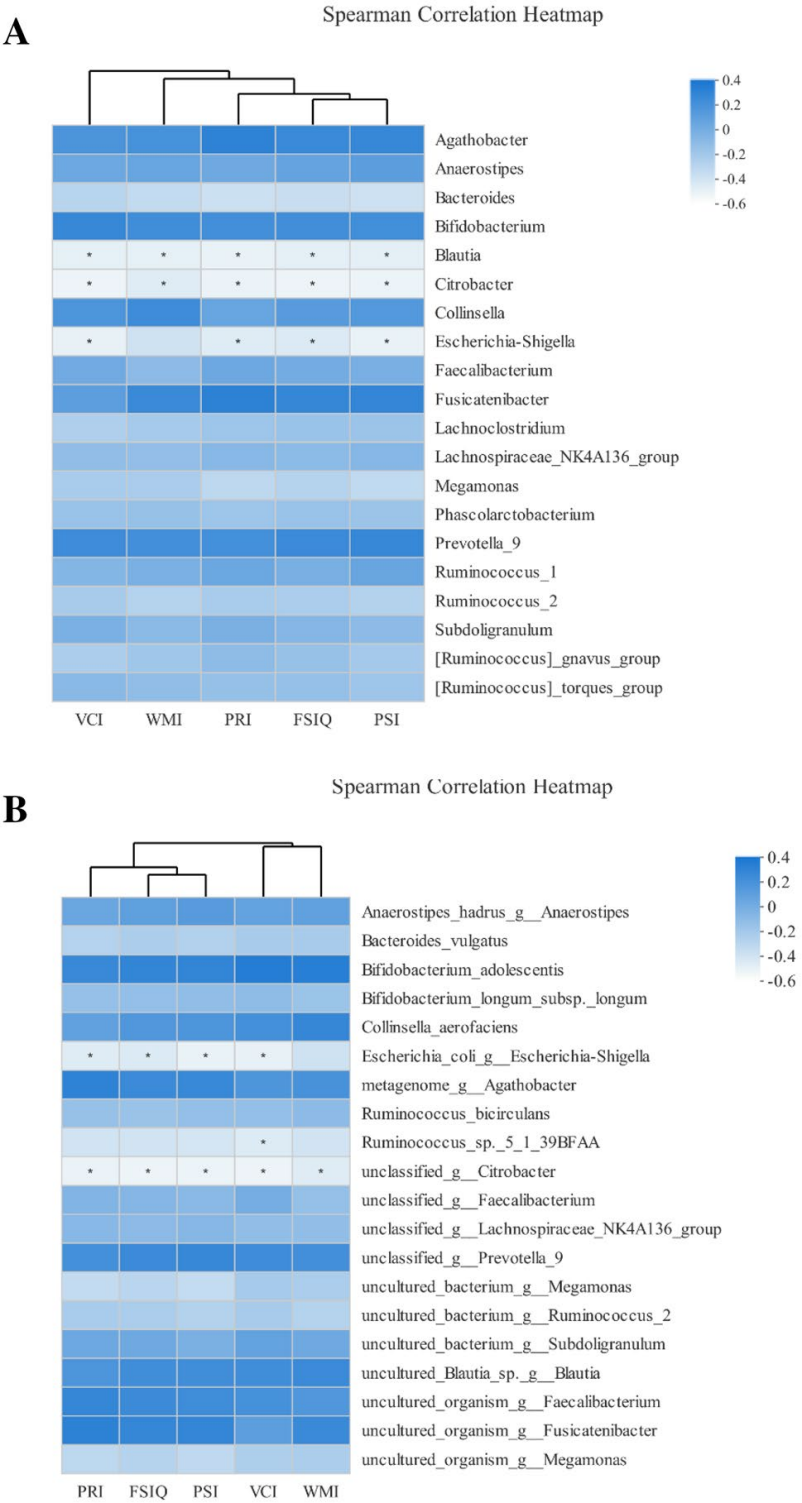
Heatmap analysis of correlation between gut microbiota composition and WISC-IV scores. The heatmap shows the correlation coefficient between bacterial taxa and the WISC-IV scores at the genus (**a**) and species (**b**) level. The legend on the right is the color interval of different *R* values. *Means 0.01 < *p* ≤ 0.05, A *p* less than 0.05 indicates the significant correlation between the WISC-IV scores and fecal microbiota

**Fig. 8:**
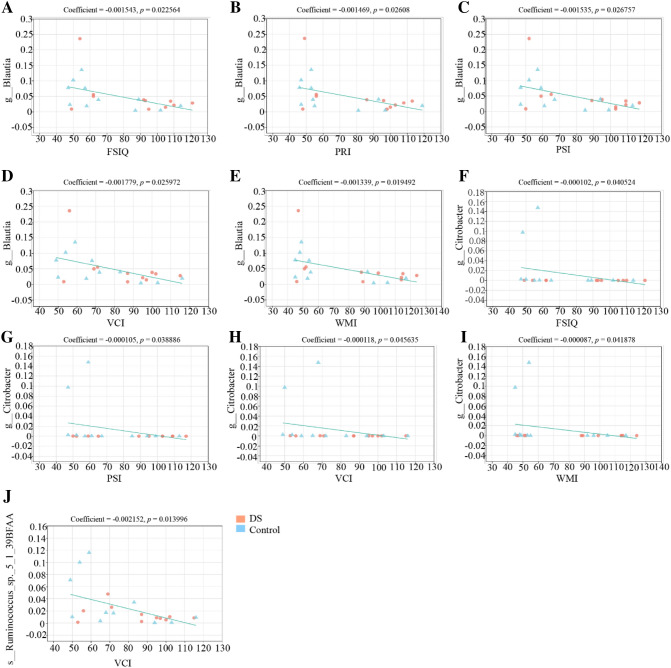
MaAslin analysis of correlation between gut microbiota composition and WISC-IV scores. The charts show the correlation coefficient between bacterial taxa and the WISC-IV scores at the genus (**a**–**i**) and species (**j**) level. The *X*-axis represents the WISC-IV score, and the *Y*-axis represents the relative abundance of bacterial taxa. Coefficient of correlation between WISC-IV scores and bacterial taxa (greater than 0 means positive correlation, less than 0 means negative correlation, equal to 0 means no correlation); The *p* value is used to measure the reliability of the test. A *p* less than 0.05 indicates a significant correlation between WISC-IV scores and bacterial taxa

The PCA on OTU level colored by FSIQ score was significantly different (*R* = 0.1186, *p* = 0.037, Fig. 6). It indicates that the fecal microbial composition of these two groups was significantly different. The interpretation degree of PC1 axis and PC2 axis to the results were 22.89% and 11.12%, respectively. Next, we examined correlations between the WISC-IV scores and fecal microbiota genera and species. All significant correlations were negative. The significant negative correlations between genus of *Blautia* and all WISC-IV scores were identified by both Heatmap analysis and MaAslin analysis (Figs. 7a, 8a–e). The significant negative correlations between genus of *Citrobacter* and FSIQ, PSI, VCI, and WMI scores were identified by both Heatmap analysis and MaAslin analysis (Figs. 7a, 8f–i). The significant negative correlations between species of *ruminococcus_sp._5_1_39BFAA* and VCI scores were identified by both Heatmap analysis and MaAslin analysis (Figs. 7b, 8j).

## Discussion

Our study confirms that gut microbiota alterations occur in Chinese children with DS and that alterations significantly correlate with cognitive function in DS. Interestingly, we found that the *Blautia* and *Citrobacter* genera were negatively associated with cognitive scores measured in our study. Importantly, the rank forest discriminating models generated in this study can effectively distinguish DS from healthy controls. Moreover, the KEGG pathway analysis indicated that the modules for ribosome biogenesis, replication and repair of chromosome, peptidases of enzyme families, and pyrimidine metabolism enriched in DS patients. To the best of our knowledge, this is one of the first studies characterizing the gut microbiota of Chinese children with DS. The recruitment of well-matched healthy controls is another important strength of our study, which greatly reduces the impacts of age, geographic region, and eating habits on gut microbiota profile outcomes. Additionally, larger cohorts that controlled for more confounders including sex, body mass index and stool consistency are needed to confirm our findings.

In our study, the Shannon diversity index of the fecal microbiota in children with DS was significantly reduced compared to the healthy group. Reduction in microbiota diversity has recently been found to be associated with autism [22], AD [23, 24], Parkinson’s disease [25], and other conditions linked to gut microbiota alterations [26, 27]. In a previous study investigating the fecal microbiota in adult DS patients using 454 pyrosequencing of the V4 region of the 16S rRNA gene, Elena Biagi et al. reported no statistically significant differences in the Shannon diversity index in Italian adult DS patients, indicating the importance of controlling for environmental factors between the DS and control groups [16]. Additionally, the beta-diversity index in Chinese children with DS differed significantly from that of healthy group, which is consistent with previous study [16]. Larger sample size studies are needed to confirm the differences in beta-diversity after accounting for stool consistency using the Bristol stool chart.

Previous comparable studies addressing differences in the gut microbiota between DS patients and controls have mainly examined the phylum or family levels [16]. Since we obtained more sequencing reads than previous studies, our results are focused on differences at the genus and species levels. One innovation of our study is the discovery of several new different taxa between DS children and well-matched healthy controls. Interestingly, only family *Acidaminococcaceae* remained statistically different after adjusting for FDR. However, the effect size of 95% confidence interval cannot strongly support obvious difference. It may be related to the limited sample size. The family *Acidaminococcaceae*, which includes the recognized genera *Acidaminococcus*, *Phascolarctobacterium*, *Succinispira* and *Succiniclasticum*, is a known producer of propionate [28]. The decreased abundance of *Acidaminococcaceae* in DS children may lead to a decrease in fecal propionate. *Ravinder* et al. have reported that fecal propionate correlates negatively with Aβ-42 in cerebrospinal fluid of subjects with mild cognitive impairment [29]. Moreover, it has been reported that the accumulation of Aβ 42 in AD brain is associated with the elevation of IL-22 and neuro-inflammation [30]. Importantly, a decreased abundance of *Acidaminococcaceae* has been found following IL-22 neutralization [31]. Overall, these results indicate that the gut microbiota in Chinese children with DS has undergone changes which may result in neuronal damage through neuro-inflammation.

The random forest model of the predominant taxa in the fecal microbiota used to identify DS in our study had similar area under the ROC curve as previous studies [22]. The KEGG pathway of ribosomal biogenesis is enriched in DS and is closely related to cognitive impairment [32, 33], Allen et al. reported that transient disruption of ribosome biogenesis could cause memory impairment [34], whereas persistent severe disruption can lead to cell death due to insufficient protein synthesis [35, 36]. Therefore, gut microbiota alterations may also affect cognitive function via the ribosomal biogenesis pathway.

It has been reported that there are significant differences in the gut microbiota among different populations and individuals due to ethnic, geographical, host genetics, age and other factors [37]. To minimize the effect of age on the microbiota, healthy controls of the same age as DS subjects were included. Each healthy control included in our study had lived in the same school as DS subjects for at least two years. This approach can largely reduce the effect of diets on the results. Similar to AD, specific taxa in the DS microbiome were correlated with cognitive function scores in this study. We found that *Citrobacter* and *Blautia* genera were negatively associated with the cognitive scores, demonstrating that the microbiota may influence cognitive function or that cognitive function may affect the microbiota [38, 39]. However, the second hypothesis has not been reported in the literature. The negative association between *Escherichia-Shigella* and WISC-IV scores, which was founded in Heatmap analysis, was not observed in the MaAslin analysis. It indicates that false positives were excluded by multiple comparative correction. In addition, there seem to be two individuals driving the correlations in MaAslin analysis. Given the small sample size, it is difficult to know whether these individuals are outliers or representative. More larger-scale studies are needed to confirm these findings. As a sulfur-producing bacterium, *Citrobacter* can produce copious amounts of hydrogen sulfide, which is the reversible inhibitor of cytochrome c oxidase. And hydrogen sulfide can influence the energy generation process mediated by the cytochrome c oxidase and cause neural damage in the brain [40, 41]. Increased abundance of *Blautia* genera has been reported in an AD mouse model [42]. And this model overexpressed the amyloid precursor protein gene, suggesting common characteristics of gut microbiota alterations between AD and DS [43]. Although an association has been reported in above animal experiments, no clinical research has directly indicated an association between cognitive impairment and the genus of *Blautia*. Our results provide the first evidence of a relationship between these bacteria taxa and DS cognitive impairment.

From our study, we confirm that gut microbiota in the DS children is altered. However, the specific role of the gut microbiota in the pathogenesis of cognitive impairment in DS cannot be clarified from this cross-sectional study. It needs more large sample size studies and further analysis tools (such as the gut-brain modules introduced by the *Raes* lab) to clarify the mechanisms involved and to explore therapeutic options for improving DS cognitive function by regulating gut microbiota [44, 45]. We hope to provide a promising strategy for future studies.

The limitations of this study should be considered. First, our study is a cross-sectional study with a limited sample size, and large-scale longitudinal studies focusing on DS patients of different age or populations are needed to confirm our results. Second, fecal samples used in microbiome analysis cannot reflect the biogeography of the microbiome across the gastrointestinal tract. This is a critical point, because the fecal microbiome which better represents the main connections to the microbiota–gut–brain axis is in the upper small intestine. Using endoscopy to collect stool samples from the upper small intestine would be a more effective method. Third, metagenomics analysis is needed to provide more detailed microbial community information, especially in terms of species function analysis and deeper analysis of DS fecal microbiota.

## Conclusion

Overall, we provide evidence of gut microbiota alterations in a Chinese cohort of children with Down’s syndrome. Additionally, the identification of genera *Blautia* and *Citrobacter* may provide a promising strategy for future studies of DS cognitive impairment. Larger cohorts from different geographic regions are needed to confirm these findings and to clarify the mechanisms involved.

## Supplementary Information

Below is the link to the electronic supplementary material.

Supplementary file1 (DOCX 767 KB)

## Data Availability

The high-throughput sequence data have been deposited in the China National Center for Bioinformation (CNCB) BioProject database (https://bigd.big.ac.cn/) with the project number of PRJCA004065. All other data are available upon request from the authors.
