# Supplementary material for: Altered gut microbiota correlates with cognitive impairment in Chinese children with Down’s syndrome
Source: Eur Child Adolesc Psychiatry. 2021 May 17;31(1):189–202. doi: 10.1007/s00787-021-01799-2 (PMC8816804; doi:10.1007/s00787-021-01799-2)
Supplement: Supplementary file 1 — Supplementary file1 (DOCX 767 KB) [file 787_2021_1799_MOESM1_ESM.docx]

**Altered Gut Microbiota Correlates with Cognitive Impairment in**

**Chinese Children with Down’s syndrome**

Shimeng Ren^a^*, Xinjuan Wang^b^*, Jiong Qin^a^, Qing Mu^b^, Shuai Ye^a^, Yang Zhang^a^, Weidong Yu^b#^, Jingzhu Guo^a#^

^a^ Department of Pediatrics, Peking University People’s Hospital, Beijing, 100044, China;

^b^ Department of Central Laboratory & Institute of Clinical Molecular Biology, Peking University People’s Hospital, Beijing 100044, China.

* Shimeng Ren and Xinjuan Wang contributed equally to this work.

Correspondence should be addressed to either of the following address:

Jingzhu Guo, E-mail: jingzhu.guo@bjmu.edu.cn;

or Weidong Yu, E-mail: [weidongyu@bjmu.edu.cn](mailto:weidongyu@bjmu.edu.cn).

**Supplementary materials**

**
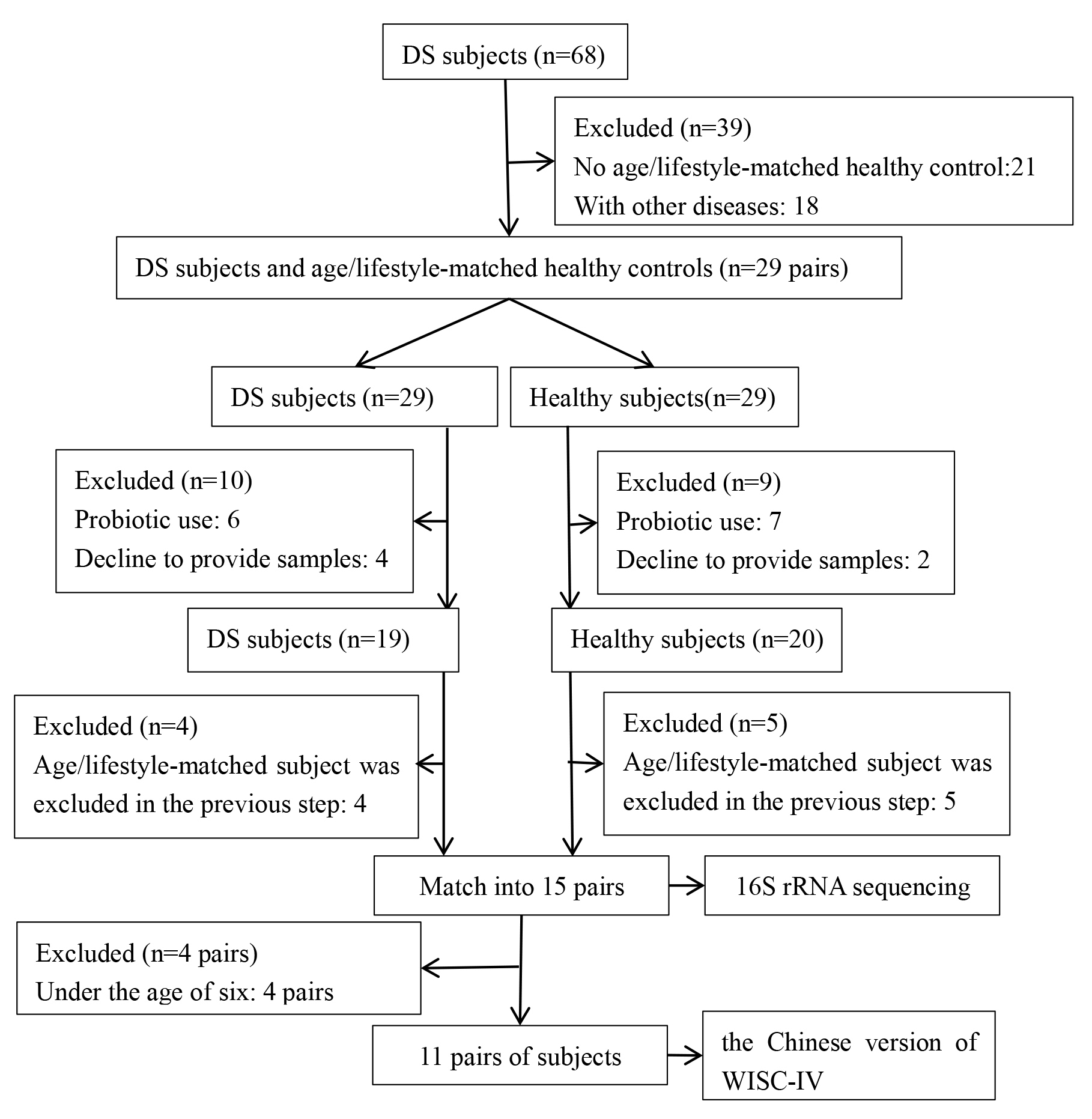
**

**Fig. S1 Detail flow chart illustrating the recruitment of DS and healthy subjects.**

**
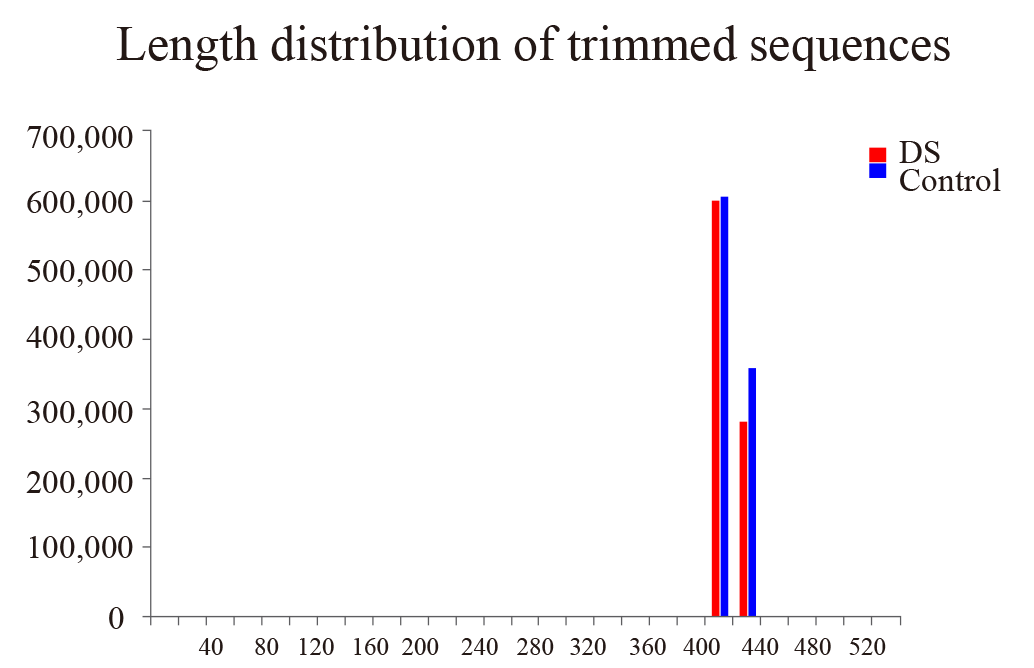
**

**Fig. S2 Length distribution of sequences in DS group and healthy control group.** The X-axis is the length interval, and the Y-axis is the number of sequences.

**
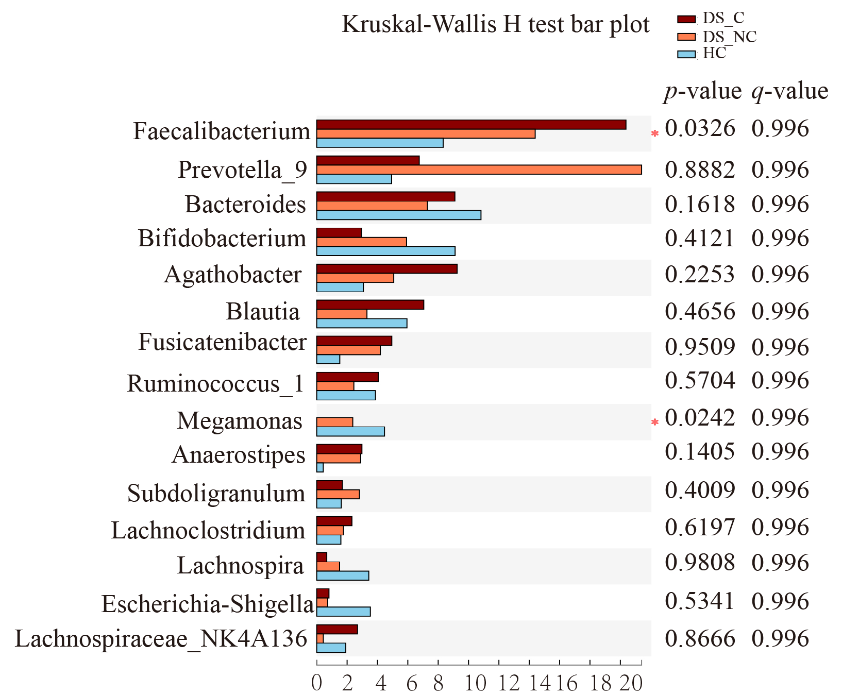
**

**
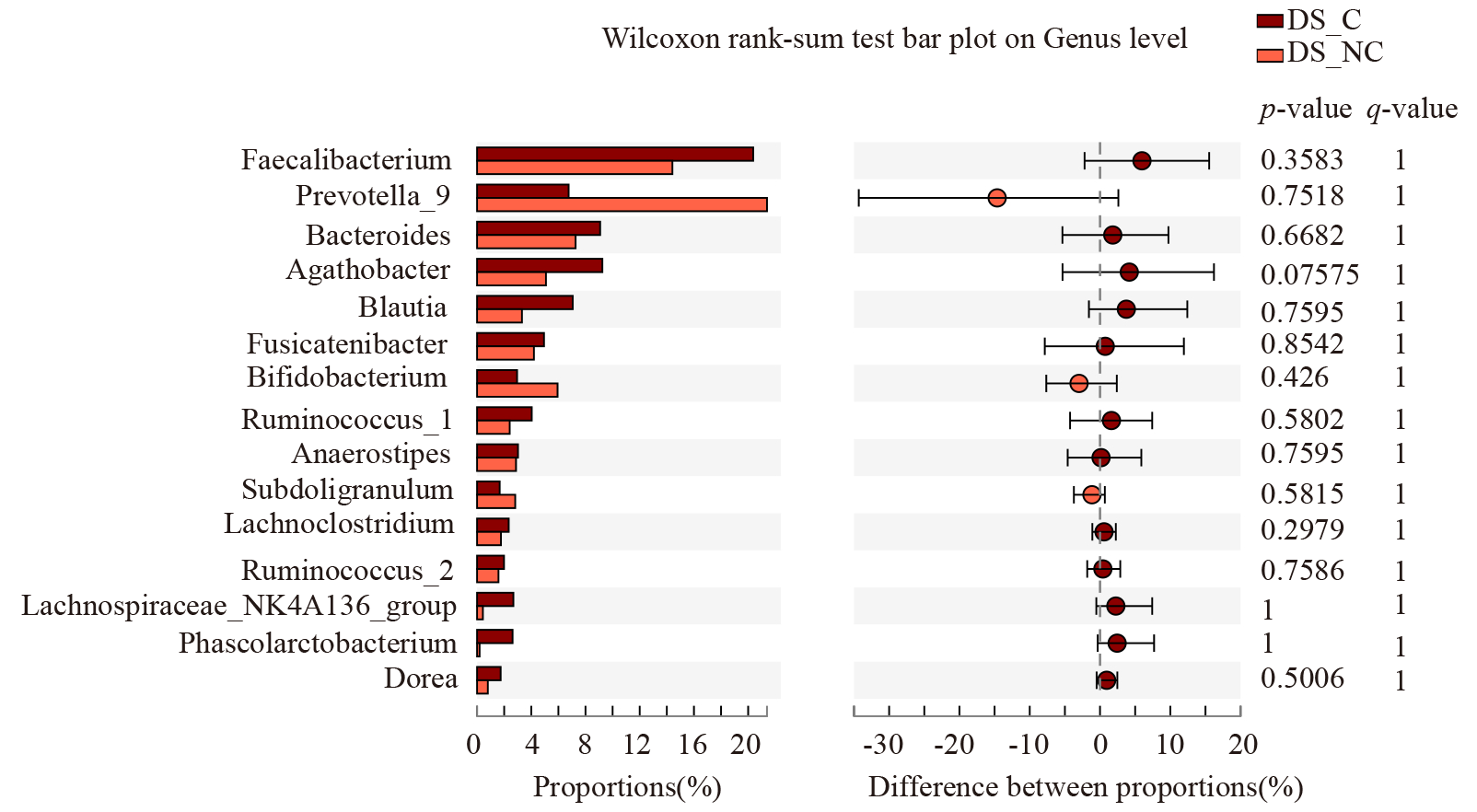
Fig. S3 Abundances of genus level in HC group, DS_C group and DS_NC group.** The Y axis represents a certain genus, the X axis represents the average relative abundance in different groups, and the columns with different colors represent different groups. Kruskal-Wallis test, the false discovery rate (FDR) was calculated using the Benjamini-Hochberg method. * 0.01 < *p* ≤ 0.05.

**Fig. S4 Abundances of genus level in DS_C group and DS_NC group.** The Wilcoxon rank-sum test at the genus level was performed to identify the differential taxa. The FDR was calculated using the Benjamini-Hochberg method.

**Supplementary Tables**

**Table S1** Differences of the fecal microbiota at all levels between DS and healthy group using Wilcoxon rank-sum test analysis

| taxonomic levels | microbiota | DS  (n=15) | Healthy control  (n=15) | *p*-value | *q*-value |
| --- | --- | --- | --- | --- | --- |
| Class | *Gammaproteobacteria* | 1.819% | 7.296% | 0.049 | 0.9504 |
| Order | *Coriobacteriales* | 0.852% | 1.865% | 0.046 | 0.9161 |
| Family | *Coriobacteriaceae* | 0.708% | 1.73% | 0.025 | 1 |
| Family | *Acidaminococcaceae* | 1.007% | 1.203% | <0.001 | 0.004 |
| Genus | *Megamonas* | 1.586% | 4.432% | 0.024 | 1 |
| Genus | *Faecalibacterium* | 16.41% | 8.357% | 0.018 | 1 |
| Genus | *Anaerostipes* | 2.932% | 0.426% | 0.046 | 1 |
| species | *Bacteroides* | 0.171% | 3.354% | 0.046 | 1 |
| species | *Anaerostipes_hardrus* | 2.922% | 0.426% | 0.026 | 1 |

*q*-value, Benjamini-Hochberg false discovery rate (FDR-P)-corrected *p* value

**Table S2** Different proportions of *Acidaminococcaceae* between DS group and Healthy control

| Group | Mean(%) | Sd(%) | *p-*value | *q*-value | Lower CI | Upper CI | Effect size |
| --- | --- | --- | --- | --- | --- | --- | --- |
| DS | 1.007 | 3.319 | 0.000389 | 0.04001 | -1.819 | 1.445 | 0.1965 |
| Healthy control | 1.203 | 0.9514 |  |  |  |  |  |

Sd, standard deviation; CI, confidence interval;

*q*-value, Benjamini-Hochberg false discovery rate (FDR-P)-corrected *p* value

**eMethods**

**16s rRNA gene amplicon and sequencing**

The isolated bacterial genomic DNA was used as a template for PCR amplification of V3-V4 region of the bacterial 16S ribosomal RNA gene in a multiplex approach with the forward primers (5′- ACTCCTACGGGAGGCAGCAG -3′) and the reverse primer (5′- GGACTACHVGGGTWTCTAAT -3′). PCR amplification was performed in 20-μL reactions containing 10X polymerase mix (Life Technologies, Carlsbad, CA, USA), 10 µM of the primers, and 25 ng of template DNA. PCR reaction parameters: 1× (3 minutes at 95°C), Number of cycles × (30 seconds at 95°C; 30 seconds at annealing temperature °C; 45 seconds at 72°C), 10 minutes at 72°C, 10°C until halted by user.

The PCR products were examined using by 2% AGE, and then gel-purified using AxyPrep DNA Gel Extraction Kit (Axygen Biosciences, Union City, CA, USA) followed by quantified with a NanoDrop ND2000 spectrophotometer (Thermo Scientific, Wilmington, DE, USA). The sequencing data were pooled equimolarly and paired-end sequenced (2×300) on an Illumina MiSeq platform (Illumina, San Diego, USA) according to standard protocols from Majorbio Bio-Pharm Technology Co. Ltd. (Shanghai, China).

**Sequence and statistical analysis**

The raw 16s rRNA gene data were processed to form operational taxonomic units (OTUs) at 97% identity using UPARSE[1]. In order to obtain the species classification information corresponding to each OTU, the Ribosomal Database Project (RDP) classifier was used for classification comparison of OTU representative sequences with 97% similar level (Taxonomic database, silva 132/16s_bacteria). The α-diversity and β-diversity indices were calculated based on the rarefied OTU counts using the Qiime program. α-diversity represents an analysis of diversity in a single sample reflected by parameters including good coverage, Chao 1, Ace, Sobs, Shannon index, and Simpson index using Qiime[2]. Wilcoxon test in R was used to compare each α-diversity index.

β-diversity is used as a measure of the microbiota structure between groups. The results of weighted Unifrac distance matrices were plotted in the principal coordinate analysis (PCoA), and analyses of similarities (ANOSIMs) were performed using the R package “ade4”.

Microorganism features used to distinguish the fecal microbiotas specific to DS were identified using the linear discriminant analysis (LDA) effect size (LEfSe) method (http://huttenhower.sph.harvard.edu/lefse/) with an effect size cutoff of 2.0.

The differential abundance analysis of taxa was performed using the Wilcoxon rank-sum test at the phylum, class, order, family, and genus levels. For multiple comparisons of bacterial counts, the false discovery rate (FDR) was calculated using the Benjamini-Hochberg method.

Phylogenetic Investigation of Communities by Reconstruction of Unobserved States (PICRUSt) was used to predict the abundances of functional categories the Kyoto Encyclopedia of Genes and Genomes (KEGG) ortholog (KO). The graph of KEGG pathways in level 2 and level 3 (http://www.genome.jp/kegg/pathway.html) was performed with STAMP, and *p* values was calculated with Wilcoxon rank-sum test, the FDR was calculated using the Benjamini-Hochberg method.

In the descriptive analyses, we used the mean and standard deviation (SD) for normally distributed continuous variables and the median and interquartile range (IQR) for continuous variables with skewed distributions.

**References**

1. Edgar RC (2013) UPARSE: highly accurate OTU sequences from microbial amplicon reads. Nat Methods 10 (10):996-998. doi:10.1038/nmeth.2604

2. Caporaso JG, Kuczynski J, Stombaugh J, Bittinger K, Bushman FD, Costello EK, Fierer N, Pena AG, Goodrich JK, Gordon JI, Huttley GA, Kelley ST, Knights D, Koenig JE, Ley RE, Lozupone CA, McDonald D, Muegge BD, Pirrung M, Reeder J, Sevinsky JR, Turnbaugh PJ, Walters WA, Widmann J, Yatsunenko T, Zaneveld J, Knight R (2010) QIIME allows analysis of high-throughput community sequencing data. Nat Methods 7 (5):335-336. doi:10.1038/nmeth.f.303
